# A potential role for macrophages in maintaining lipopolysaccharide-induced subacute airway inflammation in rats

**DOI:** 10.3892/etm.2012.726

**Published:** 2012-09-27

**Authors:** LIN LIU, LEI CHEN, YONGSHENG WANG, HUA YANG, YIFANG CHEN, XIAOYA XU, HANG ZHOU, FANGPING JIANG, TONGLIN LI, JUNLI WANG

**Affiliations:** 1 Department of Respiratory Medicine, 363 Hospital, Chengdu, Sichuan, 610041;; 2 Department of Respiratory Medicine, West China Hospital, West China School of Medicine, Sichuan University, Chengdu, Sichuan 610041;; 3 Department of Cardiology, 363 Hospital, Chengdu, Sichuan 610041, P.R. China

**Keywords:** subacute airway inflammation, lipopolysaccharide, macrophages

## Abstract

Bacterial infection is a key factor in airway inflammation. The present study describes the time-dependent changes in the leukocyte counts and cytokine levels of the bronchoalveolar lavage fluid (BALF) following subacute airway inflammation induced by lipopolysaccharide (LPS), a major component of the outer membranes of Gram-negative bacteria. LPS (200 *μ*g/rat) or saline was intratracheally administered to rats which were sacrificed 2, 4 or 7 days after LPS treatment. Airway inflammation was evaluated using hematoxylin and eosin staining, cell counts and proinflammatory cytokine levels in the BALF. Rat airways obtained from the LPS group exhibited marked airway wall thickening and infiltration of inflammatory cells compared with the control group, as well as elevated cell counts (neutrophils, macrophages, lymphocytes) and proinflammatory cytokine levels [(tumor necrosis factor (TNF)-α, interleukin (IL)-1β, cytokine-induced neutrophil chemoattractant (CINC)-1)] in the BALF, which peaked on day 2 and subsequently decreased until the experimental endpoint. Notably, IL-1β levels induced by LPS changed in a similar manner to macrophage cell counts, but not neutrophil and lymphocyte counts. Moreover, TNF-α and CINC-1 levels did not decrease as rapidly as neutrophil counts after peaking. These findings suggest that macrophages may play a significant role in maintaining subacute inflammatory responses induced by LPS in rat airways.

## Introduction

Bacterial infections, including Gram-negative bacterial infections, have been revealed to be closely involved with airway inflammatory injuries in acute exacerbations of chronic obstructive pulmonary disease (AECOPD) and asthma exacerbations (1).

Lipopolysaccharide (LPS) is a major component of the outer membranes of Gram-negative bacteria (2). LPS is recognized as an important determinant of the virulence of these organisms and the symptomatology that accompanies a Gram-negative bacterial infection. In rodent models, inhaled or instilled LPS causes acute airway and pulmonary inflammation, characterized by the recruitment of inflammatory leukocytes and the release of a variety of inflammatory mediators (3,4).

The pattern of changes in inflammatory cell counts and cytokine levels during the subacute phase of LPS-stimulated airway inflammation has not been well described. In the present study, an LPS-induced rat model was used to investigate time-course changes in subacute airway inflammation, as evidenced by airway histopathology, cell counts and proinflammatory cytokine levels in the bronchoalveolar lavage fluid (BALF).

## Materials and methods

### Animals and reagents

Male Sprague-Dawley rats (220±20 g) were purchased from the Experimental Animal Center of Sichuan University (Sichuan, China). LPS from *E. coli* serotype 055:B5 was purchased from Sigma (St. Louis, MO, USA).

### Animal treatment

A total of 30 rats were divided into a saline-treated experimental group (control group, n=15) and an LPS-treated group (LPS group, n=15). Rats were anesthetized intraperitoneally with chloral hydrate (3 ml/kg). LPS (200 *μ*g/rat) in 100 *μ*l of saline was administered by intratracheal instillation to LPS rats and the same volume of intratracheal saline was administered to control animals. Rats were sacrificed 2, 4 or 7 days after LPS or saline administration. The animal study was approved by the Committee of Laboratory Animal Care of 363 Hospital (Sichuan, China).

### Histopathology

The middle lobes of the rats’ right lungs were embedded in paraffin, following fixation in 10% buffered formalin, and then processed to obtain 4-*μ*m sections for hematoxylin and eosin (H&E) staining. The inflammatory score of H&E-stained lung sections was graded according to a previously described method (5). This scoring method strictly adhered to the blinded principle.

### BALF and cell counts

The left trachea was cannulated under deep anesthesia and an aliquot of 5 ml saline (0.9% NaCl at room temperature) was injected into the lung. Subsequently, 4.5 ml of the total volume was recovered. The recovered fluid was centrifuged at 1500 x g for 5 min to sediment the cells. After two washes with PBS solution, cells were suspended in PBS containing 10% heat-inactivated fetal calf serum and counted using a hemocytometer. Differential cell counts were determined from cell suspensions presented on slides using a cytocentrifuge (Cytospin 2; Shandon, Sewickley, PA, USA). Cells on slides were dried, fixed and then stained using the May-Giemsa method. A total of 200 cells were identified under a photomicroscope.

### Measurement of cytokines

The concentrations of tumor necrosis factor (TNF)-α, interleukin (IL)-1β and cytokine-induced neutropil chemoattractant (CINC)-1 in the BALF were measured using enzyme-linked immunosorbent assay (ELISA) kits (R&D Systems, Minneapolis, MN, USA). Samples were measured photometrically by an automated plate reader (Microplate Reader Model 1680; Bio-Rad, Hercules, CA, USA). All assays were performed in duplicate.

### Statistical analysis

The SPSS 13.0 software package (SPSS Inc., Chicago, IL, USA) was used for the statistical analyses. Values were expressed as mean ± SD. A one-way ANOVA and the Student Newman-Keuls test was used to compare the differences between the groups. P<0.05 was considered to indicate a statistically significant difference.

## Results

### Histologic changes in rat airways following LPS stimulation

**Figure 1:**
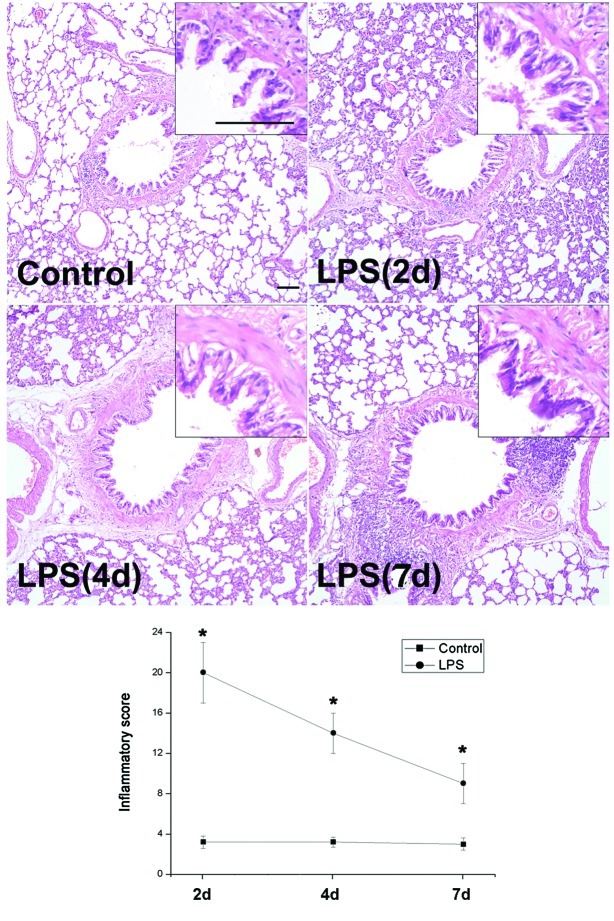
Representative microphotographs of HE-stained rat airways and inflammatory scores. Bar=100 *μ*m. ^*^P<0.05 vs. corresponding controls. LPS, lipopolysaccharide; d, days; H&E, hematoxylin and eosin.

To assess the histomorphology of rat airways following LPS stimulation, H&E staining and inflammatory scoring were performed. Marked airway wall thickening with the infiltration of inflammatory cells, which peaked on day 2, was revealed in rat airways obtained from the LPS group and began to decrease on day 4 (Fig. 1). The inflammatory scores changed in a similar manner (Fig. 1).

### Changes in cell counts and proinflammatory cytokine levels in the BALF following LPS stimulation

**Figure 2:**
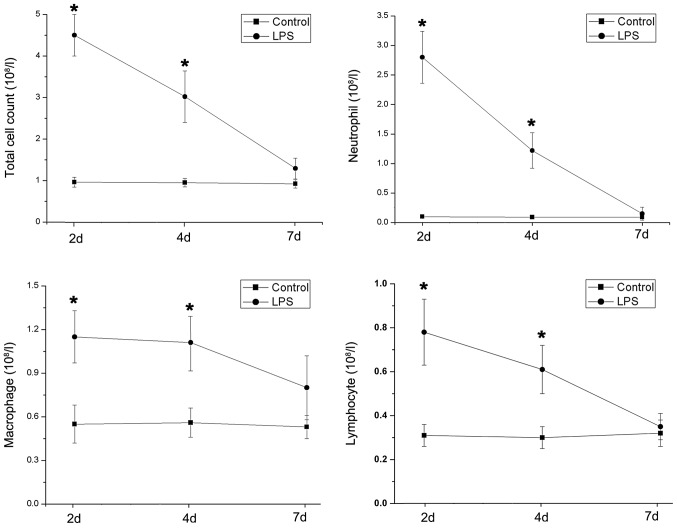
Cell counts of leukocytes in the BALF. ^*^P<0.05 vs. corresponding controls. LPS, lipopolysaccharide; d, days; BALF, bronchoalveolar lavage fluid.

**Figure 3:**
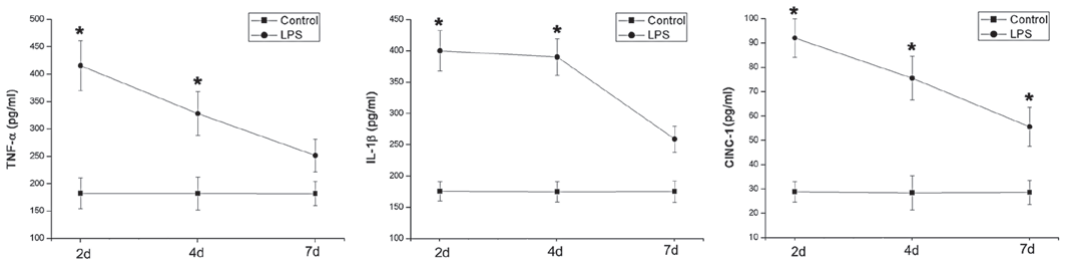
Levels of proinflammatory cytokines in the BALF. ^*^P<0.05 vs. corresponding controls. LPS, lipopolysaccharide; d, days; TNF-α, tumor necrosis factor-α; IL-1β, interleukin-1β; CINC-1, cytokine-induced neutrophil chemoattractant-1, BALF, bronchoalveolar lavage fluid.

The inflammatory level of rat airways was further evaluated using cell counts and proinflammatory cytokine levels in the BALF. The total and differential counts of inflammatory cells increased significantly following LPS treatment, although the patterns of change were not all alike (Fig. 2). Macrophages, but not neutrophils and lymphocytes, maintained a relatively stable level from day 2 to day 4 following LPS stimulation. Notably, inflammatory cytokine levels peaked on day 2 and sharply decreased until the endpoint of the study. However, the concentrations of proinflammatroy cytokines decreased less rapidly than the neutrophil count after peaking (Fig. 3).

## Discussion

In the present study, the time-course changes in leukocyte counts and cytokine levels of the BALF in LPS-induced subacute airway inflammation were described in detail. Following LPS stimulation, the proinflammatory cytokine levels and cell counts in the BALF peaked on day 2 and subsequently decreased on days 4 and 7, in accordance with the alterations to the airway histomorphology.

Following activation by stimuli (e.g., LPS), leukocytes, particularly neutrophils and macrophages, are recruited into airway lumens where they generate inflammatory mediators (6). Macrophages and neutrophils appear to have significant roles in the early airway inflammation response. Although neutrophils are considered to be the main source of proinflammatory cytokines in acute airway inflammation, macrophages are the predominant cells in the airway defence system which is the key determinant of the severity of airway inflammation (7,8). Airway macrophages not only constitute a potentially powerful source of pronflammatory and anti-inflammatory cytokines and tissue-degrading proteinases and antiproteinases, but are also involved in the removal of cells undergoing apoptosis (9*–*11). Notably, in the present study, macrophages maintained a relatively stable level from day 2 to day 4. By contrast, neutrophil and lymphocyte counts, decreased markedly after peaking on day 2. Similarly, proinflammatory cytokine levels decreased after peaking on day 2, but the evaluated cytokine levels did not decline as sharply as the neutrophil counts. IL-1β levels changed in a similar manner to macrophage counts. Together, these findings suggest that macrophages may contribute more to the maintenance of the subacute phase of LPS-stimulated airway inflammation than neutrophils.

In this process, proinflammatory cytokines play a critical role in LPS-associated subacute inflammatory reactions. An increasing number of studies have underlined the potential importance of TNF-α and IL-1β as pivotal cytokines in the initiation of the early inflammatory response to LPS exposure, although Moreland *et al* reported that a blockade of TNF-α and/or IL-1β expression was unable to protect mouse airways from acute inflammatory injury after a 4-h aerosolized LPS exposure (11). Furthermore, CINC-1 (the rat homolog of human IL-8) is one of the most important chemotactic cytokines (chemokines), inducible by proinflammatory cytokines, such as TNF-α and IL-1β. CINC-1 is involved in leukocyte transmigration into tissues, a process derived from not only leukocytic cells (monocytes, neutrophils), but nonleukocytic cells (endothelial cells, fibroblasts, epithelial cells) following LPS stimulation (12,13). Our data indicate that the levels of evaluated proinflammatory cytokines participating in the inflammatory process accurately reflect the extent of LPS-induced subacute airway inflammation. Notably, the relatively stable level of proinflammatory cytokines, particularly IL-1β, contributed to the maintanence of the subacute phase of the inflammatory response.

In summary, our data present further experimental evidence of a potentially important role for macrophages in maintaining the subacute response of LPS-induced airway inflammation which is closely involved with the release of and mediation by proinflammatory cytokines.

## References

[1] Toews GB (2005). Impact of bacterial infections on airway diseases. Eur Respir Rev.

[2] Bishop RE (2005). Fundamentals of endotoxin structure and function. Contrib Microbiol.

[3] Brass DM, Hollingsworth JW, McElvania-Tekippe E, Garantziotis S, Hossain I, Schwartz DA (2007). CD14 is an essential mediator of LPS-induced airway disease. Am J Physiol Lung Cell Mol Physiol.

[4] Chen L, Wang T, Zhang JY, Zhang SF, Liu DS, Xu D, Wang X, Chen YJ, Wen FQ (2009). Toll-like receptor 4 relates to lipopolysaccharide-induced mucus hypersecretion in rat airway. Arch Med Res.

[5] Cimolai N, Taylor GP, Mah D, Morrison BJ (1992). Definition and application of a histopathological scoring scheme for an animal model of acute *Mycoplasma pneumoniae* pulmonary infection. Microbiol Immunol.

[6] Dinarello CA (2000). Proinflammatory cytokines. Chest.

[7] Peters-Golden M (2004). The alveolar macrophage: the forgotten cell in asthma. Am J Respir Cell Mol Biol.

[8] Mangan DF, Wahl SM (1991). Differential regulation of human monocyte programmed cell death (apoptosis) by chemotactic factors and pro-inflammatory cytokines. J Immunol.

[9] Welgus HG, Campbell EJ, Bar-Shavit Z, Senior RM, Teitelbaum SL (1985). Human alveolar macrophages produce a fibroblast-like collagenase and collagenase inhibitor. J Clin Invest.

[10] Linden A, Adachi M (2002). Neutrophilic airway inflammation and IL-17. Allergy.

[11] Moreland JG, Fuhrman RM, Wohlford-Lenane CL, Quinn TJ, Benda E, Pruessner JA, Schwartz DA (2001). TNF-alpha and IL-1 beta are not essential to the inflammatory response in LPS-induced airway disease. Am J Physiol Lung Cell Mol Physiol.

[12] Mukaida N (2003). Pathophysiological roles of interleukin-8/CXCL8 in pulmonary diseases. Am J Physiol Lung Cell Mol Physiol.

[13] Kuwahara I, Lillehoj EP, Lu W, Singh IS, Isohama Y, Miyata T, Kim KC (2006). Neutrophil elastase induces IL-8 gene transcription and protein release through p38/NF-(kappa)B activation via EGFR transactivation in a lung epithelial cell line. Am J Physiol Lung Cell Mol Physiol.

